# The development and application of new crystallization method for tobacco mosaic virus coat protein

**DOI:** 10.1186/1743-422X-9-279

**Published:** 2012-11-21

**Authors:** Xiangyang Li, Baoan Song, Deyu Hu, Zhenchao Wang, Mengjiao Zeng, Dandan Yu, Zhuo Chen, Linhong Jin, Song Yang

**Affiliations:** 1 State Key Laboratory Breeding Base of Green Pesticide and Agricultural Bioengineering, Key Laboratory of Green Pesticide and Agricultural bioengineering of Ministry of Education, Guizhou University, Huaxi District, Guiyang 550025, Guizhou Province, P. R China

**Keywords:** GST-tags, His-tags, Peptides, Disk form, Protein crystals, TMV-CP, Truncated protein

## Abstract

**Background:**

Although tobacco mosaic virus (TMV) coat protein (CP) has been isolated from virus particles and its crystals have grown in ammonium sulfate buffers for many years, to date, no one has reported on the crystallization of recombinant TMV-CP connecting peptides expressed in *E. coli*.

**Methods:**

In the present papers genetically engineered TMV-CP was expressed, into which hexahistidine (His) tags or glutathione-S-transferase (GST) tags were incorporated. Considering that GST-tags are long peptides and His-tags are short peptides, an attempt was made to grow crystals of TMV-CP cleaved GST-tags (WT-TMV-CP_32_) and TMV-CP incorporated His-tags (WT-His-TMV-CP_12_) simultaneously in ammonium sulfate buffers and commercial crystallization reagents. It was found that the 20S disk form of WT-TMV-CP_32_ and WT-His-TMV-CP_12_ did not form high resolution crystals by using various crystallization buffers and commercial crystallization reagents. Subsequently, a new experimental method was adopted in which a range of truncated TMV-CP was constructed by removing several amino acids from the N- or the C-terminal, and high resolution crystals were grown in ammonium sulfate buffers and commercial crystallization reagents.

**Results:**

The new crystallization method was developed and 3.0 Å resolution macromolecular crystal was thereby obtained by removing four amino acids at the C-terminal of His-TMV-CP and connecting six His-tags at the N-terminal of His-TMV-CP (TR-His-TMV-CP_19_). The Four-layer aggregate disk structure of TR-His-TMV-CP_19_ was solved. This phenomenon showed that peptides at the C-terminus hindered the growth of high resolution crystals and the peptides interactions at the N-terminus were attributed to the quality of TMV-CP crystals.

**Conclusion:**

A 3.0 Å resolution macromolecular crystal of TR-His-TMV-CP_19_ was obtained and the corresponding structure was solved by removing four amino acids at the C-terminus of TMV-CP and connecting His-tags at the N-terminus of TMV-CP. It indicated that short peptides influenced the resolution of TMV-CP crystals.

## Background

Tobacco mosaic virus (TMV) has a rod-like appearance and consists of a single, positive strand RNA of 6395 nucleotides encapsulated in a helical virion by approximately 2130 identical coat protein (CP) subunits [1-7]. CP consists of 158 amino acids that were assembled into four main alpha-helices joined by a prominent loop proximal to the axis of the virion [8-16]. TMV-CP played an important role in the self-assembly of TMV through an initial RNA recognition reaction that triggers the assembly, it was believed to be necessary for virus assembly initiation and elongation [8,17-24]. The biological physical properties of TMV-CP were often determined by the structure of TMV. The TMV structure reported in 1986 was studied based on an electron density map at 3.6 Å resolution by X-ray fiber diffraction [9], and then this structure of the complete virus was determined at 2.9 Å resolution by X-ray fiber diffraction methods [25]. TMV-CP assembly systems, consisting of 34-subunit aggregate of TMV-CP crystallized as a dimer of bilayer disks having 17 subunits per layer, were crystallized and solved at 2.8 Å resolution [2]. The crystalline structure of the Four-layer aggregate of TMV-CP was determined at 2.4 Å resolution by using X-ray diffraction from crystals maintained at cryogenic temperatures. This structure emphasized the importance of water in biological macromolecular assemblies [22,23]. The circular permutants of TMV-CP were crystallized and solved by molecular replacement at 3.0 Å resolution by using X-ray diffraction [26]. The structure of TMV was also obtained by using high resolution transmission electron microscopy [27-29].

TMV-CP was usually propagated and isolated from *Nicotiana tabacum (N. tabacum)* infected by TMV, and TMV-CP existed as a number of aggregates, depending on PH, ionic strength, temperature, protein concentration, and other factors [5,12,14-21]. At 0.1 mol/L ionic strength orthophosphate solution and pH equal to or greater than 8.0, TMV-CP existed as protein A or 4S protein (a dynamic equilibrium between monomers, trimers, and pentamers of TMV-CP). At 0.1 mol/L ionic strength orthophosphate solution and pH near 7.0, TMV-CP was transformed into the 20S aggregate form (disks consisting of 34 monomers, also named as the 20S structure) with an admixture of 4S protein [30-33]. At 0.1 mol/L ionic strength orthophosphate solution and pH equal to or less than 6.0, TMV-CP was completely transformed into macroscopic aggregate. The 34-subunit disk was virtually crystallized at 1.0 mol/L ionic strength solution and PH near 8.0 [2,3,12,17,19-21,30,31]. The crystals of TMV-CP consisting of Four-layer aggregates were usually obtained at 3.5–14 mg/mL protein concentration (10 mmol/L orthophosphate, pH 7.2) and in crystallization pool solutions consist of 0.2–0.3 mol/L ammonium sulfate and 0.1 mol/L Tris at pH 8.0 for 3 wk to 4 wk at room temperature [23]. The crystals were obtained after been equilibrated overnight against a crystallization pool solution consisting of 0.3 mol/L ammonium sulfate and 0.1 mol/L Tris at pH 8.0 by microdialysis method [23,31-36]. The aformentioned method of obtaining TMV-CP from TMV will take investigators a lot of work and time, and the TMV-CP structures obtained from TMV were aggregate forms [32-36], they could not be easily modified. In this sense, by employing the method of genetically engineered, recombination TMV-CP was expressed, purified and crystallized [37-43].

In the present investigation, the genetically engineered structure of TMV-CP was the concern: a series of the recombinant expression vectors contained TMV-CP genes were constructed and transformed into *E.coli*, and the recombinant protein of TMV-CP were expressed, and 3.0 Å resolution TR-His-TMV-CP_19_ (incorporated His-tags at the N-terminus of TMV-CP and truncated four amino acids at the C-terminus of TMV-CP) macromolecular crystals were obtained.

## Results and Discussions

### Identification to Recombinant Vectors

**Figure 1:**
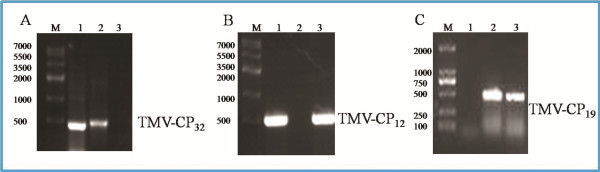
**TMV-CP DNA fragments (1% agarose gel). **(**A**) The whole TMV-CP fragments with BamH I and Xho I restriction enzyme cutting sites which have been cloned in PGEX-6P-1. As shown in lane 1, amplified PCR product ran at approximately 500 bp compared with the DNA marker (lane M). Lane 2 is a positive control with DNA template. Lane 3 is a negative control without DNA template. (**B**) The whole TMV-CP fragments with Nde I and Xho I restriction enzyme cutting sites that have been cloned in pET28a. As shown in lane 1, amplified PCR product ran at approximately 500 bp compared with the DNA marker (lane M). Lane 2 is a negative control without DNA template. Lane 3 is a positive control with DNA template. (**C**) The truncation of four amino acids from the C-terminus of TMV-CP fragments with Nde I and Xho I restriction enzyme cutting sites that have been cloned in pET28a. Lane 1 is a negative control without DNA template, whereas lane 2 is a positive control with DNA template. Lane 3 is the amplified PCR product that ran at approximately 500 bp compared with the DNA marker (lane M).

TMV-RNA has been isolated from TMV particles (propagated in *N. tabacum* K_326_) and reverse transcribed into cDNA by primer_cDNA_ and reverse transcriptase (TaKaRa). The genetic fragment of wild type TMV-CP (WT-GST-TMV-CP_32_, with the restriction enzymes of BamH I/Xho I; WT-His-TMV-CP_12_, with the restriction enzymes of Nde I/Xho I; ) were amplified by using cDNA as template. The following series of genetic fragments, four amino acids truncated at the C-terminus of WT-His-TMV-CP_12_, (TR-His-TMV-CP_19_), three amino acids truncated at the N-terminus of WT-His-TMV-CP_12_ and four amino acids truncated at the C-terminus of WT-His-TMV-CP_12_ (TR-His-TMV-CP_62_), three amino acids truncated at the N-terminus of WT-His-TMV-CP_12_, and five amino acids truncated at the C-terminus of WT-His-TMV-CP_12_ (TR-His-TMV-CP_68_), were also amplified by PCR. Compared with the DNA marker, all the PCR products migrated as expected at approximately 500 bp, and the PCR products were marked as the WT-GST-TMV-CP_32_ (Figure1A), WT-His-TMV-CP_12_ (Figure 1B), TR-His-TMV-CP_19_ (Figure1C), TR-His-TMV-CP_62_, and TR-His -TMV-CP_68_ separately.

**Figure 2:**
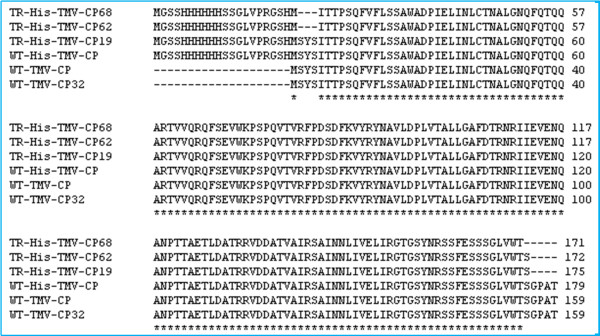
Alignment of the TMV-CP Sequences, the identical residues were marked below by an asterisk.

The corresponding clones were sequenced by ABI Automatic DNA Sequence Machine, and the correct sequences were obtained and aligned (Figure 2). The DNA sequences of WT-GST-TMV-CP_32_, WT-His-TMV-CP_12_, TR-His-TMV-CP_19_, TR-His-TMV-CP_62_, and TR-His-TMV-CP_68_ were similar that of WT-TMV-CP (isolated from TMV), except for the presence of short peptides incorporated at the N-terminal of TMV-CP in their DNA sequences. These correct proteins were successfully cloned to the expression host, *E.coli BL*_*21*_*(DE*_*3*_*)-RIL* (TakaRa), for protein expression.

### Confirmation of the Proteins Expressed and Purified by Gel Filtration

**Figure 3:**
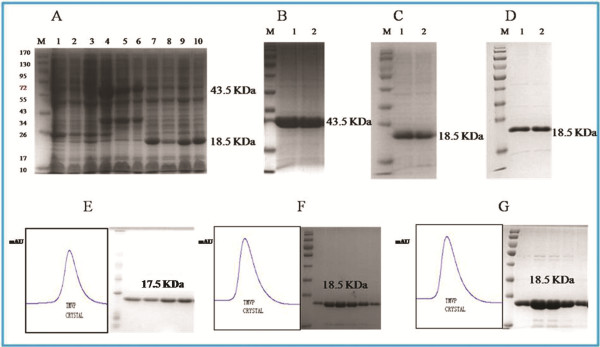
**The generation of expression and purification of target protein that was analyzed using 12% SDS-PAGE. **(**A**) Protein molecular weight standards are shown in lane 1. Numbers on the left are the MW of the standards in kDa. Lanes 1, 2, and 3 show the controls without induction by IPTG that were 20 μL aliquots of whole cell lysates from 10 mL PGEX-6P-1-WT-GST-TMV-CP_32_-*BL*_*21*_*(DE*_*3*_*)-RIL*, pET28a-His-TMV-CP_12_-*BL*_*21*_*(DE*_*3*_*)-RIL*, and pET28a-TR-His-TMV-CP_19_-*BL*_*21*_*(DE*_*3*_*)-RIL *cultures, respectively. Lanes 4, 5, and 6 correspond to the cultures after expression that were from 20 μL aliquots of whole cell lysates from 1 L PGEX-6P-1-WT-GST-TMV-CP_32_-*BL*_*21*_*(DE*_*3*_*)-RIL *cultures with IPTG. A new protein band at 43.5 kDa corresponds to the target protein GST-TMV-CP. Lanes 7 and 8 correspond to the cultures after expression that were from 20 μL aliquots of whole cell lysates from 1 L pET28a-TR-His-TMV-CP_12_-*BL*_*21*_*(DE*_*3*_*)-RIL* and pET28a-TR-His-TMV-CP_68_-*BL*_*21*_*(DE*_*3*_*)-RIL *culture with IPTG, respectively. A new protein band at position 18.5 kDa corresponds to the target protein. Lanes 9 and 10 correspond to the cultures after expression that were from 20 μL aliquots of whole cell lysates from 1 L pET28a-TR-His-TMV-CP_62_-*BL*_*21*_*(DE*_*3*_*)-RIL *and pET28a-TR-His-TMV-CP_19_-*BL*_*21*_*(DE*_*3*_*)-RIL *cultures with IPTG, respectively. A new protein band at position 18.5 kDa corresponds to the target protein. (**B**) WT-GST-TMV-CP_32_ protein is shown in lanes 1 and 2 purified using nickel-nitrilotriacetic acid (Ni-NTA) column. (**C**) WT-His-TMV-CP_12_ protein is shown in Lanes 1 and 2 purified using Ni-NTA column. (**D**) TR-His-TMV-CP_19_ protein is shown in Lanes 1 and 2 purified using Ni-NTA column. (**E**) WT-TMV-CP_32_ protein cleaved GST-tags is shown in 12% SDS-PAGE gel filtration purified using HiLoad 16/60 Superdex 200 pg column. (**F**) WT-His-MV-CP_12_ protein is shown in 12% SDS-PAGE gel filtration purified using HiLoad 16/60 Superdex 200 pg column. (**G**) TR-His-MV-CP_19_ protein is shown in 12% SDS-PAGE gel filtration purified using HiLoad 16/60 Superdex 200 pg column.

Expressed proteins of WT-GST-TMV-CP_32_, WT-His-TMV-CP_12_, TR-His-TMV-CP_19_, TR-His-TMV-CP_62_, and TR-His-TMV-CP_68_ were initially assayed by Coomassie brilliant blue method in a small scale experiment in which the final volume was 10 mL(Figure 3A).The protein products including the whole cell lysates and the target proteins were confirmed by 12% sodium dodecyl sulfate (SDS) polyacrylamide gel electrophoresis (PAGE). The molecular mass of WT-GST-TMV-CP_32_ was test by migration at approximately 43.5 kDa (Figure 3B); the molecular mass of WT-His-TMV-CP_12_ (Figure 3C), TR-His-TMV-CP_19_ (Figure 3D), TR-His-TMV-CP_62_, and TR-His-TMV-CP_68_ were test by in the same migration approximately at 18.5 kDa. The molecular mass of WT-TMV-CP_32_ (WT-GST-TMV-CP_32_ cleaved GST-tags) was test by migration at approximately 17.5 kDa (Figure 3E), and the molecular mass of TR-His-TMV-CP_12_ (Figure 3F) and TR-His-TMV-CP_19_ (Figure 3G) were test by in the same migration at approximately 18.5 kDa.

### Disk state of TR-His-TMV-CP_19_ in Solution

In the size exclusion chromatography, the retention volume of N-His-TMV-CP_19_ protein under 20 mM Sodium Phosphate buffer and 100 mM Sodium Chloride solution (PH 8.0) was the oligomeric state (dimmers and monomers), and the retention volume of TR-His-TMV-CP_19_ was transformed to disks mostly after dialyzing against 0.2–0.3 mol/L ammonium sulfate and 0.1 mol/L Tris ( PH 8.0 ) solution at room temperature for more than 10 hr. The disk state of TR-His-TMV-CP_19_ was confirmed by the map of SEC (Figure 6A) and Native-PAGE simultaneously (Figure 6B and Figure 6C).

### Identification and Diffraction of Crystals

**Figure 4:**
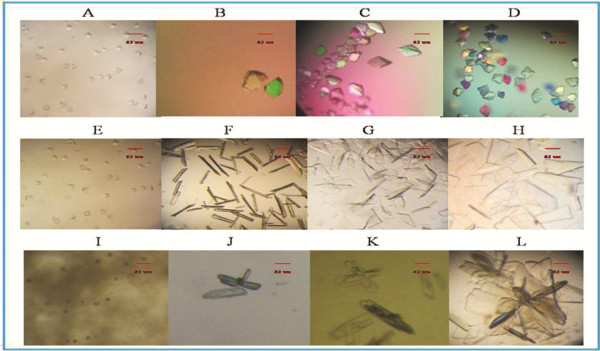
**Crystallization of WT-TMV-CP**_**32 **_**(Cleaved GST-tags) and WT-His-TMV-CP**_**12**_, **the scale bar represents 0.1 mm. **(**A**) and (**E**) Typical octahedral WT-His-TMV-CP_12_ crystals grown in the crystallization room at 295 K. The crystals did not grow bigger regardless of the time of exposure in the crystallization reagent. (**B**), (**C**), and (**D**) Screening and optimization of WT-His-TMV-CP_12_ crystallization. Although the size of the crystals improved, the quality of the crystals did not. Conversely, most of the improved crystals have no diffraction. (**F**), (**G**), and (**H**) Optimization of crystallization reagents facilitated growth of WT-His-TMV-CP_12_ crystals. Results showed that increased salt and ionic strength increased by the crystallization reagent changed the crystallization from octahedral crystals to bar and lamellar crystals. (**I**) WT-TMV-CP_32_ microcrystal that was cloned using the vector of PGEX-6P-1, purified using His-tags, cleaved GST-tags by PreScission Protease. In addition**,** seeding tools were used and crystallization reagents were changed, including the crystallization reagents of Hampton research, in an attempt to improve the quality and size of the crystals or to produce a different crystal form. Only twin crystals or polycrystalline were obtained, as shown in (**J**), (**K**), and (**L**).

Macromolecular crystals were grown in crystallization buffers with high levels of supersaturation, often reaching several hundred percent. WT-TMV-CP_32_, WT-His-TMV-CP_12_, TR-His-TMV-CP_62_, TR-His-TMV-CP_68_ crystals (Figure 4) were cultured by using Index Screen (Hampton research) and ammonium sulfate buffers. These crystals were optimized by seeding method, but high resolution crystals were not obtained.

**Figure 5:**
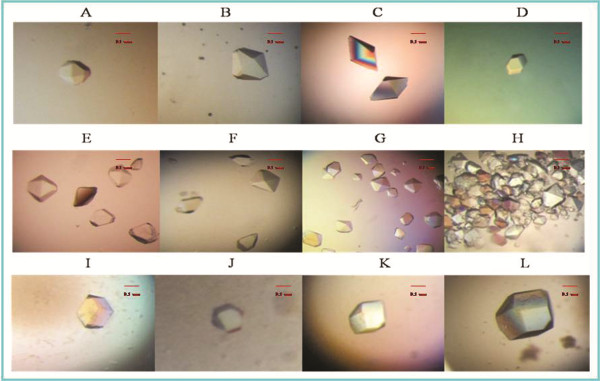
**Examples of TR-His-TMV-CP**_**19 **_**crystals with fused short peptides and truncated four amino acids from the C-terminus (Showed in Table**4**), the scale bar represents 0.1 mm**.

**Figure 6:**
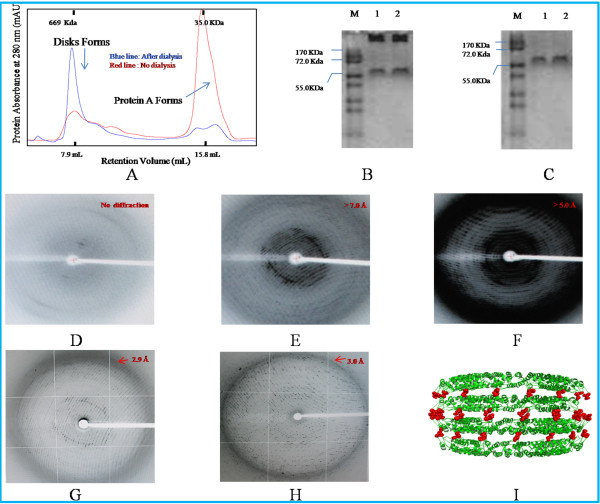
**The form of TR-His-TMV-CP19 proteins were analyzed by SEC and 17% Native-PAGE and the diffraction analysis of crystals on proteins of TR-His-TMV-CP19 and WT-His-TMV-CP12 (A) The assembly of TR-His-TMV-CP19 was measured by Superdex 200 10/300 GL Column, Blue line represented TR-His-TMV-CP19 dialyzed against 0.2 mol/L ammonium sulfate and 0.1 mol/L Tris solution ( PH 8.0 ) at room temperature for 12 hr. **Protein peaks were monitored by UV absorbance at wavelength of 280 nm and retention volumes corresponding to molecular mass were recorded in 20 mM PB and 100 mM sodium chloride, the molecular mass standards were indicated on top of the figure;Red line represents TMV-CP control which did not dialyze against, **(B)** the 17% Native-PAGE of WT-His-TMV-CP19. As shown in lane M. Numbers on the left were the MW of the standards in kDa, Lanes 1, 2, correspond to the WT-His-TMV-CP19 dialyzed against 0.2 mol/L ammonium sulfate and 0.1 mol/L Tris solution ( PH 8.0 ) at room temperature for 12 hr, **(C) **the 17% Native-PAGE of WT-His-TMV-CP19. As shown in lane M. Numbers on the left were the MW of the standards in kDa, Lanes 1, 2, correspond to the WT-His-TMV-CP19 did not dialyze against the corresponding solution,** (D**) X-ray crystal diffraction of WT-His-TMV-CP12 marked in Figure 5B, **(E**) X-ray crystal diffraction of TR-His-TMV-CP19 marked in Figure 6A, **(F)** X-ray crystal diffraction of TR-His-TMV-CP19 marked in Figure 6G,** (G**) X-ray crystal diffraction of TR-His-TMV-CP19 marked in Figure 6J,** (H**) X-ray crystal diffraction of TR-His-TMV-CP19 obtained from the conditions of Figure 6J, **(I) **The Four-layer aggregate structure of TR-His-TMV-CP19 incorporated His-tags.

On the contrary, TR-His-TMV-CP_19_ crystals (Figure 5)(protein concentration: 14 mg/mL) with 3.0 Å resolution were obtained in 0.25 mol/L ammonium sulfate and 0.1 mol/L Tris solution at 295 K, PH 7.7 (Figure 6), and the Four-layer aggregate disk structure of TR-His-TMV-CP_19_ was solved.

TMV-CP has been available as a recombinant protein expressed in *E.coli* for more than 20 years [37,38]. The incorporation of His-tags at the C-terminal of TMV-CP has been reported recently by introducing His-tags into TMV-CP to facilitate their purification [39]. To date, however, no one has reported on the crystallization of recombinant TMV-CP connecting peptides expressed in *E. coli,* except on the residues of chemical modification of TMV-CP [26,40-43]. These recombinant proteins connecting with peptides often did not affect the biological activity of the engineered proteins, located at the exterior of the TMV-CP disks. To obtain high resolution crystals, the expression vectors containing TMV-CP fragments were first constructed and expressed. An attempt was made to harvest the WT-His-TMV-CP_12_ fragments by using thrombin cleavage to cleave His-tags. His-tags were not cleaved when the proportion of thrombin cleavage and recombinant proteins was increased from 1:1 to 8:1. Subsequently, another genetically engineered WT-GST-TMV-CP_32_ was constructed and expressed. Compared with His-tags, the GST-tags were easily cleaved by PreScission Protease. The crystals of WT-TMV-CP_32_ and WT-His-TMV-CP_12_ were grown in ammonium sulfate buffers and commercial crystallization reagents simultaneously. No high resolution crystals were formed in the hanging drops when the proportion of WT-TMV-CP_32_ to crystallization solution was 1:1. Only tiny octahedral WT-His-TMV-CP_12_ crystals were grown in the crystallization room at 295 K. No matter how long the growth time was, the crystals did not grow bigger in the crystallization buffers and commercial crystallization reagents. To maintain the physical properties of WT-His-TMV-CP_12_ according to the crystalline structure of WT-TMV-CP (the amino acids at the terminal of WT-TMV-CP were flexibly) (PDB codes 1EI7 and 1VTM), three kinds of WT-His-TMV-CP_12_ truncated amino acids at the C-terminal (TR-His-TMV-CP_19_, TR-His-TMV-CP_62_, TR-His-TMV-CP_68_) were constructed, expressed, purified and formed 20S disks. And then the crystals of TR-His-TMV-CP_19_ appeared when the protein concentration (20 mmol/L orthophosphate and 100 mmol/L sodium chloride, pH 8.0, at 297 K for 12 hr) is 8.0–14 mg/mL and crystallization pool solution (pH 7.7) consisting of 0.1 mol/L ammonium sulfate and 0.1 mol/L Tris at 295 K for 24 hr. The increasing of the ionic strength (1.0) and pH of the crystallization pool buffers resulted in the appearance of TR-His-TMV-CP_19_ crystals in the circumstance of higher protein concentration. Compared with WT-TMV-CP, the purified protein of TR-His-TMV-CP_19_ was in the form of protein A (Figure 6A).

**Figure 7:**
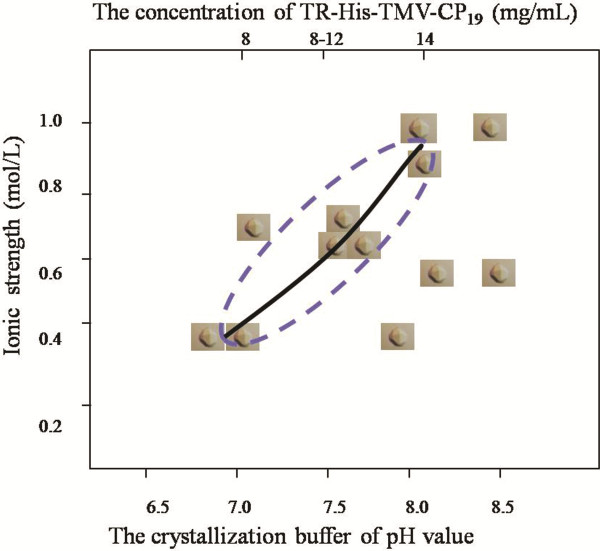
**Diagram showing the growth curve of the crystallization of TR-His-TMV-CP**_**19**_**. **The predominant species at protein concentration conditions of 8.0–14 mg/mL at pH 6.8–7.8 with ionic strength of 0.4–1.0 are the perfect crystals, identified as higher resolution crystals. As the pH falls below pH 7.0, TR-His-TMV-CP_19 _did not grow in the form of crystals. On the contrary, the TR-His-TMV-CP_19 _crystals grew with increasing protein concentration, pH (pH 7.8–8.0), and ionic strength (0.7–1.0) of the crystallization pool buffers. The crystals can also be harvested when the protein concentration is 14 mg/mL because high resolution crystals are formed at this concentration. When the crystallization pool buffers conditions are at pH 8.5 with ionic strength of 0.7–1.0, no high resolution crystals can be harvested.

The crystals of TR-His-TMV-CP_19_ were obtained at 2.9–7.0 Å resolution (Figure 6E-6H) and the crystal of WT-His-TMV-CP_12_ was obtained without diffraction. A Four-layer aggregate crystal structure of TR-His-TMV-CP_19_ was obtained by removing four amino acids at the C-terminal of His-TMV-CP and connecting short peptides at the N-terminal of His-TMV-CP (TR-His-TMV-CP_19_). A diagram of the growth curve of TR-His-TMV-CP_19_ crystallization was drawn (Figure 7).

Compared with the crystals of WT-TMV-CP (isolated from TMV particles), the Propagation, purification and acquisition the macromolecular crystals of TR-His-TMV-CP_19_ from *E.coli* were very facile process. By investigating, it was found that the crystals of WT-His-TMV-CP_12_ had close relationships with protein concentration, ionic strength and PH of the solution. After truncating four amino acids at the C-terminal of WT-His-TMV-CP_12_, a Four-layer aggregate structure of TR-His-TMV-CP_19_ was determined at 3.0 Å resolution by using the technique of Hanging-drop vapor diffusion and seeding methods, but the high resolution crystals of WT-TMV-CP_32_, WT-His-TMV-CP_12_, TR-His-TMV-CP_62_, and TR-His-TMV-CP_68_ did not obtain.

## Conclusions

The crystallization and stability of recombinant WT-TMV-CP were influenced by various factors such as the charge, isoelectric point, hydrophilicity of hybrid CP, and especially the length of the amino sequence. Based on the crystallization methods of WT-TMV-CP (isolated from TMV particles), the crystals of TR-His-TMV-CP (TR-His-TMV-CP_19_, TR-His-TMV-CP_62_, TR-His-TMV-CP_68_) were grown in the crystallization buffers as mentioned above. The crystals of TR-His-TMV-CP_19_ were grown by the technique of Hanging-drop vapor diffusion and seeding methods with crystallization reagents including several kinds of ammonium sulfate and Tris solutions. A map of the growth curve of the crystallization of TR-His-TMV-CP_19_ was drawn. In this map, the proteins of 20s disk form of TR-His-TMV-CP_19_ (8.0–14 mg/mL) were firstly prepared in solutions (pH 8.0) containing 20 mmol/L orthophosphate and 100 mmol/L sodium chloride and then dialyzed against 0.2–0.3 mol/L ammonium sulfate and 0.1 mol/L Tris solutions ( PH 8.0 ) at 277 K for 12 hr. The corresponding crystals were grown in crystallization pool solutions containing 0.1–0.3 mol/L ammonium sulfate and 0.1 mol/L Tris at pH 6.5–8.5 by the technique of Hanging-drop vapor at 277 and 295 K. Seeding methods were performed at 295 K after 1–7 d, the good macromolecular crystals appeared under different conditions. 2.9–7.0 Å resolution of TR-His-TMV-CP_19_ (concentration 14 mg/mL) macromolecular crystals were obtained in crystallization pool solution consisting of 0.25 mol/L ammonium sulfate and 0.1 mol/L Tris at pH 7.7. Then, using the same method, the crystals of WT-His-TMV-CP_12_ without resolution and some tiny WT-TMV-CP_32_ crystals, were also obtained by series of seeding experiments. It was showed from the experiment that: the genetically engineered proteins of TR-His-TMV-CP^19^ could grow high resolution crystals. Hence, the present investigations suggest that the C-terminal of TMV-CP was unstable for crystallization buffer, and the amino acids at the C-terminus were hypothesized to be very flexible. Additionally, the inserted sites of short peptides of TMV-CP could access the grown crystals. Short peptides have a positive influence on the stability of the biophysical properties of TMV-CP. Compared with WT-TMV-CP isolated from TMV particles, this recombinant protein of TR-His-TMV-CP^19^ is easy to purify and grow crystals. Thus, the latter can be applied to structural biology and structure-based drug design.

## Methods

TMV (common strain) was isolated from *N. tabacum* K_326_ leaves infected by TMV, which were cultivated in the Greenhouse of Center for Research and Development of Fine Chemicals of Guizhou University*,* and purified by the method described by Gooding [44], and modified by Shire [16,45]. TMV-CP was prepared by Scheel [18]. TMV-RNA was extracted from purified virus by treating with phenol and SDS [46-48]. In order to obtain the generation of full-length viral cDNA sequence, TMV-RNA was reverse transcribed using primer_cDNA_ (Table 1) in 50 mmol/L Tris at pH 8.0, 8.0 mmol/L magnesium chloride, 75 mmol/L potassium chloride, 10 mmol/L DL-Dithiothreitol, 1.0 mmol/L dNTPs, 0.5 unit/μL AMV reverse transcriptase (TaKaRa), and 1.0 unit/μL RNase inhibitor (TaKaRa) for 1.5 hr at 315 K.

**Table 1:** DNA sequences of the primers

**serial number**	**Name**	**Primers**
Primer_cDNA_	TMV-cDNA	5’-TCGACATAGGGACATCTTC-3’
Primer_1_^**a,c**^	WT-TMV-CP-FOR	5’-GGAATTCCATATGTCTTACAGTATCACTACTCC-3’
Primer_2_^**b,d**^	WT-TMV-CP-REV	5’- CCGCTCGAGTCAAGTTGCAGGACCAGAGG-3’
Primer_3_^**b**^	TR-TMV-CP-FOR	5’-CGGGATCCATGTCTTACAGTATCACTACTCC-3’
Primer_6_^**a,d,e**^	TR-His-TMV-CP-FOR	5’-GGAATTCCATATGATCACTACTCCATCACAGTTCG-3’
Primer_8_^**e**^	TR-His-TMV-CP-REV	5’-CCGCTCGAGTCAACCAGAGGTCCAAACCAA-3’
Primer_9_^**c**^	TR-His-TMV-CP-REV	5’-CCGCTCGAGTCAAGTTGCAGGACCAGAGG-3’

^**a**^The WT-His-TMV-CP_12 _fragment was amplified by using primer_1_ and primer_2_.

^**b**^The WT-GST-TMV-CP_32 _fragment was amplified by using primer_3_ and primer_2_.

^**c**^The TR-His-TMV-CP_19 _fragment was amplified by using primer_1_and primer_9_.

^**d**^The TR-His-TMV-CP_62 _fragment was amplified by using primer_6_ and primer_2_.

^**e**^The TR-His-TMV-CP_68 _fragment was amplified by using primer_6_ and primer_8_.

Using the generation of full-length viral cDNA sequence and the corresponding primers by the same method, TR-His-TMV-CP_19_, WT-GST-TMV-CP_32_, WT-His-TMV-CP_12_, TR-His-TMV-CP_62_, and TR-His-TMV-CP_68_ sequences were amplified by PCR technology (Table 2).

**Table 2:** Protein building blocks used or referenced herein

**Building block**	**Origin**	**Abbreviation**
Wild type TMV coat protein	*N. tabacum *K_326_	WT-TMV-CP^a^
Wild type TMV coat protein with GST tags	*E. coliBL*_*21*_*(DE*_*3*_*)-RIL*	WT-GST-TMV-CP_32_^b^
Wild type TMV coat protein with His tags	*E. coliBL*_*21*_*(DE*_*3*_*)-RIL*	WT-His-TMV-CP_12_^c^
Truncated 19 TMV coat protein with His tags	*E. coliBL*_*21*_*(DE*_*3*_*)-RIL*	TR-His-TMV-CP_19_^d^
Truncated 62 TMV coat protein with His tags	*E. coliBL*_*21*_*(DE*_*3*_*)-RIL*	TR-His-TMV-CP_62_^e^
Truncated 68 TMV coat protein with His tags	*E. coliBL*_*21*_*(DE*_*3*_*)-RIL*	TR-His-TMV-CP_68_^f^

^**a **^The wild type TMV-CP was purified from *N. tabacum *K_326_.

^**b**^The wild type TMV-CP expressed and purified from reconstructed prokaryotic expression vector PGEX-6P-1-TMV-CP_32 _that was expressed in competent cell *BL*_*21*_*(DE*_*3*_*)-RIL*.

^**c**^The wild type TMV-CP expressed and purified from reconstructed prokaryotic expression vector pET28a-TMV-CP_12 _that was expressed in competent cell *BL*_*21*_*(DE*_*3*_*)-RIL*.

^**d**^The truncated TMV-CP expressed and purified from reconstructed prokaryotic expression vector pET28a-TMV-CP_19 _that was expressed in competent cell *BL*_*21*_*(DE*_*3*_*)-RIL*_._

^**e**^The truncated TMV-CP expressed and purified from reconstructed prokaryotic expression vector pET28a-TMV-CP_62 _that was expressed in competent cell *BL*_*21*_*(DE*_*3*_*)-RIL*_._

^**f**^The truncated TMV-CP expressed and purified from reconstructed prokaryotic expression vector pET28a-TMV-CP_68 _that was expressed in competent cell *BL*_*21*_*(DE*_*3*_*)-RIL.*

The dsDNA of correct length was purified and identified by 1% agarose gel electrophoresis. Both plasmid pET28a (Novagen) and CP were digested with Nde I (NEB, 10 units/μL)/Xho I (NEB, 10 units/μL) and cloned into the same sites in pET28a (pET28a-WT-His-TMV-CP_12_, pET28a-TR-His-TMV-CP_19_, pET28a-TR-His-TMV-CP_62_, and pET28a-TR-His-TMV-CP_68_). Both plasmid PGEX-6P-1 (Novagen) and CP were digested with BamH I (NEB, 10 units/μL)/Xho I (NEB, 10 units/μL) and cloned into the same sites in PGEX-6P-1 (PGEX-6P-1-WT-GST-TMV-CP_32_). Transcription reactions were performed by using the corresponding transcription system. *E. coli BL*_*21*_*(DE*_*3*_*)-RIL* (TaKaRa) cultures were transformed into vectors involving aforementioned recombinant plasmid. Expression plasmids were grown in Luria-Bertani (LB) medium containing 30 μg/mL kanamycin at 310 K until the OD_600_ reached 0.65–1.0. After cooling the cultures to 289 K, the expression product was induced by the addition of 1.0 mmol/L IPTG, and the culture was incubated for 16 hr. The cells were harvested by centrifugation and resuspended in 35 mL lysis buffer (100 mmol/L sodium chloride, 50 mmol/L phosphate buffer, pH 8.0, 10 mmol/L *ß*-mercaptoethanol). Then, the cells were thawed, lysed by supersonic device, and then centrifuged at 15000 rpm for 30 min at 277 K. The supernate was then passed through 0.22 mm syringe filters (Millipore) and loaded onto a Ni Sepharose High performance column (GE Healthcare, 5mL), washed with five column volumes of 40 mmol/L imidazole, and eluted with 400 mmol/L imidazole. The N-terminal His-tags failed to cleaved with thrombin (1.0 unit/mg) and N-terminal GST-tags was cleaved successfully with PreScission Protease (1.0 unit/mg) by incubating overnight at 277 K. The cleaved GST-tags and uncleaved His-tags were removed by the same chelating column, and the flow-through was concentrated in an Amicon Ultra centrifugal filter device (Millipore) with a 10 kDa filter and then loaded onto a HiLoad 16/60 Superdex 200 pg column equilibrated in the dialysis solution (20 mmol/L orthophosphate and 100 mmol/L sodium chloride, pH 8.0). The protein was concentrated to 5.0–25 mg/mLfor the crystallization trials by using Amicon Ultra centrifugal filter units (Millipore) with a 10 kDa molecular weight cutoff. The target proteins were briefly stored at 277 K.

The purification proteins were dialyzed against the appropriate high-salt solution at room temperature to obtain the Four-layer aggregate (20S disk) [35,36,49-52]. The 20S disk form proteins were confirmed by Size Exclusion Chromatography (SEC) and Native-polyacrylamide gel Electrophoresis (Native-PAGE) method. SEC was performed at room temperature by using a calibrated Superdex 200 10/300 GL column (GE Healthcare) attached to an AKTApurifier fast protein liquid chromatography system (GE Healthcare). The column was equilibrated with a solution containing 20 mM orthophosphate (pH 8.0), 100 mM NaCl solution. Molecular mass standards (Bio-Rad) used are: Thyroglobulin (669 kDa), Ferritin (440 kDa), BSA (67 kDa), *β*-lactoglobulin (35 kDa), Ribonuclease A (13.7 kDa), Cytochrome (13.6 kDa), Aprotinin (6.51 kDa) and Vitamin B12 (1.36 kDa). Protein was monitored by absorbance at the wavelength of 280 nm. The crystals of purified proteins were obtained by the technique of Hanging-drop vapor diffusion. The protein concentration was 5.0–25 mg/mL, and the crystallization solutions contained 0.1–0.3 mol/L ammonium sulfate and 0.1 mol/L Tris at PH 6.5–8.5 (Table 3) for 1–7 d at 293–298 K. The crystals (Table 4) were first soaked with cryoprotection (the reservoir solution containing an extra 30% glycerol), and then mounted and flash-frozen in liquid nitrogen [33,34]. Diffraction data were collected at Shanghai Synchrotron Radiation Facility beamline 17U. All the X-ray data were processed by using HKL2000 program suite and converted to structure factors within the CCP4 program (Table 5).

**Table 3:** The crystallization solutions of ammonium sulfate

**Sample**	**Protein concentration** ** and buffer**^**a**^	**Crystallization ** **buffer**^**b**^	**Temperature** **(K)**
WT-TMV-CP_32_ (cleaved GST)	8 –14 mg/mL, 20 mmol/L sodium phosphate buffer, 100 mmol/L sodium chloride, pH 8.0	0.10–0.35 mol/L ammonium sulfate, 0.1 mol/L Tris buffer, pH 6.5–8.5	277/295
WT-His-TMV-CP_12_	8–14 mg/mL, 20 mmol/L sodium phosphate buffer, 100 mmol/L sodium chloride, pH 8.0	0.10–0.35 mol/L ammonium sulfate, 0.1 mol/L Tris buffer, pH 6.5–8.5	277/295
TR-His-TMV-CP_19_	8–14 mg/mL, 20 mmol/L sodium phosphate buffer, 100 mmol/L sodium chloride, pH 8.0	0.10–0.35 mol/L ammonium sulfate, 0.1 mol/L Tris buffer, pH 6.5–8.5	277/295
TR-His-TMV-CP_68_	8–14 mg/mL, 20 mmol/L sodium phosphate buffer, 100 mmol/L sodium chloride, pH 8.0	0.10–0.35 mol/L ammonium sulfate, 0.1 mol/L Tris buffer, pH 6.5–8.5	277/295
TR-His-TMV-CP_62_	8–14 mg/mL, 20 mmol/L sodium phosphate buffer, 100 mmol/L sodium chloride, pH 8.0	0.10–0.35 mol/L ammonium sulfate, 0.1 mol/L Tris buffer, pH 6.5–8.5	277/295

^**a**^ The buffers were provided with filter membrane.

^**b**^ The buffers were provided with filter membrane.

**Table 4:** **Examples of TR-His-TMV-CP**_**19 **_**crystals in Figure**5

**Examples**	**Protein Concentration**	**Crystal Conditions**	**Crystal appearance**	**Resolutions**
A	8.0 mg/mL (20 mmol/L sodium phosphate buffer, 100 mmol/L sodium chloride, pH 8.0)	0.1 mol/L ammonium sulfate and 0.1 mol/L Tris/HCl, pH 6.8	The typical dodecahedral crystals were rapid growth (approximately 24 hr) at 295 K	>7.0 Å
B	8.0 mg/mL (20 mmol/L sodium phosphate buffer, 100 mmol/L sodium chloride, pH 8.0)	0.1 mol/L ammonium sulfate and 0.1 mol/L Tris/HCl, pH 7.5.	The irregular octahedral crystals were rapid growth (approximately 24 hr) at 295 K	--
C	8.0 mg/mL (20 mmol/L sodium phosphate buffer, 100 mmol/L sodium chloride, pH 8.0)	0.1 mol/L ammonium sulfate and 0.2 mol/L Tris/HCl, pH 7.0.	The irregular octahedral crystals were rapid growth (approximately 24 hr) at 295 K	--
D	8.0 mg/mL (20 mmol/L sodium phosphate buffer, 100 mmol/L sodium chloride, pH 8.0)	0.3 mol/L ammonium sulfate and 0.1 mol/L Tris/HCl, pH 7.7.	The typical octahedral crystals were rapid growth (approximately 24 hr) at 295 K	--
E	8.0 mg/mL (20 mmol/L sodium phosphate buffer, 100 mmol/L sodium chloride, pH 8.0)	0.2 mol/L ammonium sulfate and 0.1 mol/L Tris/HCl, pH 6.8.	The octahedral crystals were rapid growth (approximately 24 hr) at 295 K	--
F	8.0 mg/mL (20 mmol/L sodium phosphate buffer, 100 mmol/L sodium chloride, pH 8.0)	0.2 mol/L ammonium sulfate and 0.1 mol/L Tris/HCl, pH 7.7.	The octahedral crystals were rapid growth (approximately 24 hr) at 295 K	--
G	8.0 mg/mL (20 mmol/L sodium phosphate buffer, 100 mmol/L sodium chloride, pH 8.0)	0.2 mol/L ammonium sulfate and 0.1 mol/L Tris/HCl, pH 6.8.	The Octahedral crystals were rapid growth (approximately 24 hr) at 295 K	>5.0 Å
H	14 mg/mL (20 mmol/L sodium phosphate buffer, 100 mmol/L sodium chloride, pH 8.0)	0.3 mol/L ammonium sulfate and 0.1 mol/L Tris/HCl, pH 8.0.	The splintered off and crowded together crystals were rapid growth (approximately 24 hr) at 295 K	--
I	22 mg/mL (20 mmol/L sodium phosphate buffer, 100 mmol/L sodium chloride, pH 8.0)	0.3 mol/L ammonium sulfate and 0.1 mol/L Tris/HCl, pH 7.7.	The hexahedral octahedral crystals were Slow growth (approximately 7 d) at 295 K	--
J	14 mg/mL (20 mmol/L sodium phosphate buffer, 100 mmol/L sodium chloride, pH 8.0)	0.25 mol/L ammonium sulfate and 0.1 mol/L Tris/HCl, pH 7.7.	The typical octahedral crystals were Slow growth (approximately 1-2 d) at 295 K	2.9–3.0 Å
K	22 mg/mL (20 mmol/L sodium phosphate buffer, 100 mmol/L sodium chloride, pH 8.0)	0.2 mol/L ammonium sulfate and 0.1 mol/L Tris/HCl, pH 7.7.	The dodecahedral crystals were slow growth (approximately 24 hr) at 295 K	--
L	22 mg/mL (20 mmol/L sodium phosphate buffer, 100 mmol/L sodium chloride, pH 8.0)	0.2 mol/L ammonium sulfate and 0.1 mol/L Tris/HCl, pH 7.7.	The dodecahedral crystals were Slow growth (approximately 9d) at 295 K	--

- stand for the crystals which didn't been diffraction by X-ray methods.

**Table 5:** **Data collection and refinement statistics of TR-His-TMV-CP**_**19 **_**crystals in Figure**6I

	**TR-His-TMV CP**_**19**_
**Data collection**	
Space group	P2_1_2_1_2
Cell dimensions	
*a*, *b*, *c *(Å)	171, 311, 314
α, β, γ (°)	90, 90, 90
Resolution (Å)	50.0-3.06 (3.17-3.06)
*R*_sym_	0.11 (0.196)
*I*/σ(*I)*	10.1 (6.4)
Completeness (%)	99.7 (99.2)
Redundancy	4.4 (3.84)
**Refinement statistics**	
Resolution (Å)	20.0-3.06 (3.14-3.06 )
No. reflections	155016
*R*_work_/*R*_free_	20.4/25.5
No. atoms	36918
B-factors	47.9
R.m.s deviations	
Bond lengths (Å)	0.013
Bond angles (º)	1.499

*Highest resolution shell is shown in parenthesis.

## Abbreviations

TMV: Tobacco mosaic virus; CP: Coat protein; His: Hexahistidine; GST: Glutathione-S-transferase; Ni-NTA: Nickel-nitrilotriacetic acid; dsDNA: Double-stranded DNA; cDNA: Complementary DNA; IPTG: Isopropyl *ß*-D-thiogalactopyranose; SDS: Dodecylsulfate; PAGE: Polyacrylamide gel electrophoresis; Native-PAGE: Native-polyacrylamide gel Electrophoresis; SEC: Size Exclusion Chromatography; WT: Wild type; TR: Truncation; Tris: Tris(hydroxymethyl)aminomethane; DTT: DL-dithiothreitol; LB: Luria-Bertani; *E.coli*: *Escherichia coli*; *N. tabacum*: *Nicotiana tabacum*.

## Competing interests

The authors declare that they have no competing interests.

## Authors’ contributions

BAS and SY designed this study. XYL carried out the clone and protein expression studies, participated in the sequence alignment and drafted the manuscript. ZC,ZCW,MJZ and DDY performed Crystallization tests. LHJ collected and analyzed the data of genetically engineered TMV-CP crystals. BAS, SY, DYH, and ZC critically revised the manuscript. All of the authors read and approved the final version of the manuscript.
